# Potential Chemopreventive Activity of a New Macrolide Antibiotic from a Marine-Derived *Micromonospora* sp

**DOI:** 10.3390/md11041152

**Published:** 2013-04-03

**Authors:** Skylar Carlson, Laura Marler, Sang-Jip Nam, Bernard D. Santarsiero, John M. Pezzuto, Brian T. Murphy

**Affiliations:** 1 Department of Medical Chemistry & Pharmacognosy, College of Pharmacy, University of Illinois at Chicago, 833 S. Wood Street, Chicago, IL 60612, USA; E-Mails: scarls21@uic.edu (S.C.); bds@uic.edu (B.D.S.); 2 College of Pharmacy, University of Hawaii at Hilo, 34 Rainbow Drive, Hilo, HI 96720, USA; E-Mails: lmarler@hawaii.edu (L.M.); pezzuto@hawaii.edu (J.M.P.); 3 Department of Chemistry and Nano Science, Ewha Womans University, Seoul 120-750, Korea; E-Mail: freenam75@gmail.com

**Keywords:** macrolide, *Micromonospora*, quinone reductase 1, chemoprevention, actinomycete, marine

## Abstract

Agents capable of inducing phase II enzymes such as quinone reductase 1 (QR1) are known to have the potential of mediating cancer chemopreventive activity. As part of a program to discover novel phase II enzyme-inducing molecules, we identified a marine-derived actinomycete strain (CNJ-878) that exhibited activity with cultured Hepa 1c1c7 cells. Based on this activity, a new macrolide, juvenimicin C (**1**), as well as 5-*O*-α-l-rhamnosyltylactone (**2**), were isolated from the culture broth of a *Micromonospora* sp. Compound **1** enhanced QR1 enzyme activity and glutathione levels by two-fold with CD values of 10.1 and 27.7 μM, respectively. In addition, glutathione reductase and glutathione peroxidase activities were elevated. This is the first reported member of the macrolide class of antibiotics found to mediate these responses.

## 1. Introduction

Cancer initiation is the result of subcellular damage from electrophilic metabolites of exogenous carcinogens and endogenous reactive oxygen species (ROS) [1]. Quinone reductase 1 (QR1) is a phase II enzyme responsible for two-electron reduction and detoxification of such metabolites. Induction of this enzyme has been shown to be a biomarker for chemoprevention [2]. Accordingly, secondary metabolites capable of inducing this enzyme can be used to slow the process of carcinogenesis. 

Several studies report QR1-inducing compounds of both semi-synthetic and natural product origins. This suite of compounds exhibits QR1 doubling concentrations (CD) in the mM to nM range. Some common QR1-inducing structural classes include stilbenes and tetrahydro-β-carbolines. In regard to the latter, semi-synthetic efforts significantly improved QR1 CDs, reaching values as low as 0.2 μM through addition of alkylated *N*-urea derivatives to a core piperidine ring system [3]; this was an improvement on bioactivity observed from naturally occurring derivatives (e.g., perlolyrin, CD = 1.7 μM) [4]. Similarly, the QR1 inducing activity of the stilbene resveratrol (CD = 21 μM) was enhanced to submicromolar levels through generation of thiazole linked analogues (CD values ranging from 0.087 to 0.98 μM) [5]. This bioactivity was comparable to that of the most potent inducer discovered to date, 4′-bromoflavone (CD = 0.1 μM) [6].

A few other classes of secondary metabolites have been shown to induce QR1 activity such as triterpenes [7], flavonoids [8,9,10,11], labdane diterpenes [12], and other small molecular weight phenolic compounds [4,13], though the majority of these structures exhibit moderate to weak CD values when compared to their stilbene, flavonoid, and β-carboline counterparts.

As part of a program to identify molecules that induce the expression of QR1, screening of an actinomycete secondary metabolite fraction library led to the selection of strain CNJ-878 for further investigation on the basis of bioactivity in QR1 enzyme assays. In the current study, we present the first report of a macrolide antibiotic, juvenimicin C (**1**), that induces QR1 and other phase II detoxifying enzymes with moderate potency. Details of the structure elucidation and biological activities are described herein. 

## 2. Results and Discussion

### 2.1. Structure Elucidation

**Figure 1:**
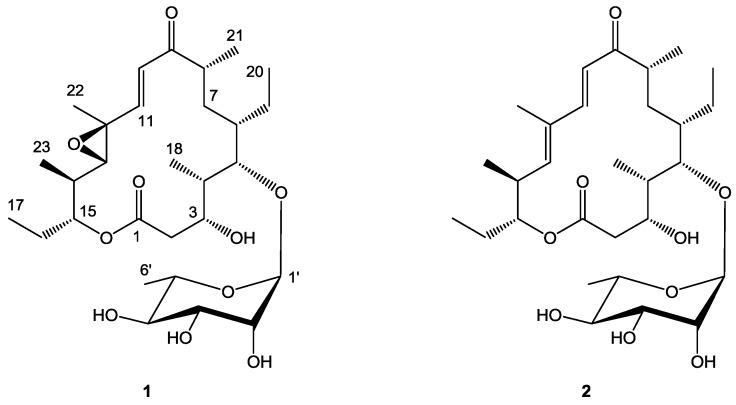
Structure of juvenimicin C (**1**) and 5-*O*-α-l-rhamnosyltylactone (**2**).

**Figure 2:**
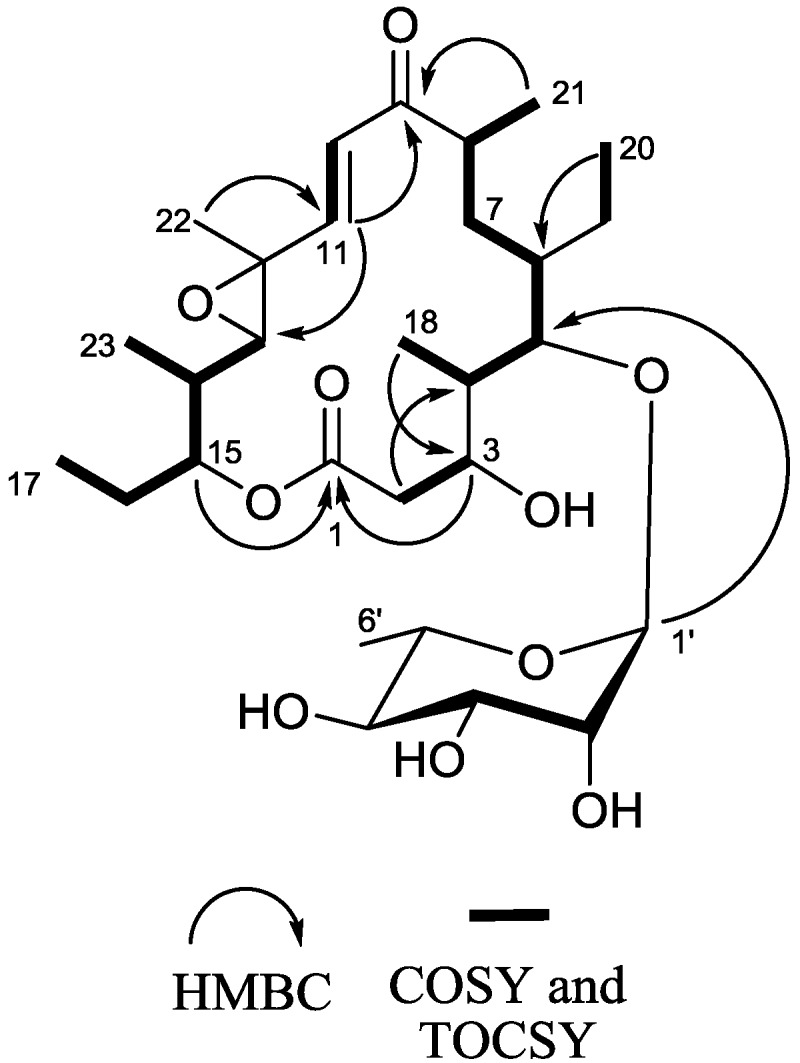
Key 2D NMR correlations of **1**.

After several rounds of chromatography, juvenimicin C (**1**; Figure 1) was obtained as white powder. The molecular formula of **1** was assigned as C_29_H_48_O_10_ on the basis of combined NMR and MS experiments. This formula demanded six degrees of unsaturation. Analysis of HMBC and HSQC NMR experiments suggested the presence of an α,β-unsaturated ketone carbonyl (δ_C_ 202.4, C-9), two olefinic carbons (δ_C_ 124.8, C-10; 150.5, C-11), two epoxide carbons (δ_C_ 60.7, C-12; 68.8, C-13), and an ester functional group (δ_C_ 174.8, C-1). There was evidence of eight *sp*^3^ oxygenated carbons (δ_C_ 67.4, C-3; 84.7, C-5; 77.7, C-15; 72.0, C-2′; 72.3, C-3′; 73.3, C-4′; 69.8, C-5′), one of which was identified as an anomeric carbon (δ_C_ 104.0, C-1′) (Table 1). Given that the molecular formula afforded six degrees of unsaturation and the molecule contained one sugar, an α,β-unsaturated ketone, an epoxide, and an ester group, the remaining degree was satisfied by the macrolide ring system. Key HMBC, COSY, and TOCSY correlations are given in Figure 2. Interpretation of COSY data defined six spin systems, which were then connected to each other using HMBC correlations. 

**Table 1:** ^1^H and ^13^C NMR data (600, 150 MHz, CD_3_CN) of **1**.

	1	
Pos	^13^C	^1^H mult. ( *J*, Hz)
1	174.8	
2	40.4	2.59 dd (9.0, 10.2)
		2.19 d (9.0)
3 Eq	67.4	3.69 d (10.2)
4	41.6	1.75 m ^a^
5	84.7	3.58 d (9.6)
6	39.6	1.14 m
7	33.3	1.76 m
		1.38 m
8	46.0	2.62 m
9	202.4	
10	124.8	6.65 d (15.9)
11	150.5	6.34 d (15.9)
12	60.7	
13	68.8	2.80 d (9.6)
14	38.4	1.75 m ^a^
15	77.7	4.83 dt (10.0, 2.4)
16	25.1	1.80 m
		1.49 m
17	9.3	0.88 d (7.2)
18	10.1	0.95 d (6.6)
19	22.5	1.50 m
		1.36 m
20	12.5	0.87 d (7.2)
21	17.5	1.16 d (6.6)
22	15.3	1.43 s
23	14.5	1.08 d (6.6)
1′	104.0	4.62 br s
2′	72.0	3.89 br s
3′	72.3	3.47 d (7.2)
4′	73.3	3.29 m
5′	69.8	3.63 m
6′	17.5	1.18 d (6.0)
3-OH		3.22 br s

^a^ Resonances are overlapping.

An HMBC correlation from H_2_-2 to C-4 positioned fragment C-2–C-3 next to the spin system of C-18, C-4, C-5, C-6, C-7, C-8, and C-21. A COSY correlation was observed from H_3_-20 to H_2_-19, though no correlation was observed from H_2_-19 to H-6; thus an HMBC correlation from H_3_-20 to C-6 helped solidify the position of the ethyl substituent. An HMBC correlation from H_3_-21 to C-9 suggested the α,β-unsaturated ketone was adjacent to the aforementioned spin system. A *J*-3 correlation from H-11 to C-9 further supported the position of the α,β-unsaturated carbonyl moiety, the chromophore of which was observed in the UV spectrum of **1**. An HMBC correlation from H-11 to C-13 linked the epoxide to the α,β-unsaturated system, while COSY connectivities placed a five-carbon unit (H_3_-23, H-14, H-15, H_2_-16, and H_3_-17) adjacent to C-13. Finally, an HMBC correlation connected the oxygenated methine H-15 to the ester carbonyl at C-1 to complete the flat macrolide skeleton. 

Additional signals were present in the ^1^H NMR spectrum that could not be attributed to the macrolide aglycone. Subtracting those atoms accounted for by the macrolide, the remaining fragment had a formula of C_6_H_11_O_5_. Resonances typical of an anomeric carbon (δ_C_ 104.0 and δ_H_ 4.62) were observed in both ^1^H NMR and HMBC experiments suggesting the macrolide was glycosylated. Five of the eight observed oxygenated carbons were connected via COSY and TOCSY experiments to afford the fragment C-1′–C-6′. The macrolide was glycosylated at C-5, as evidenced by an HMBC correlation from the anomeric proton H-1′ to C-5.

**Figure 3:**
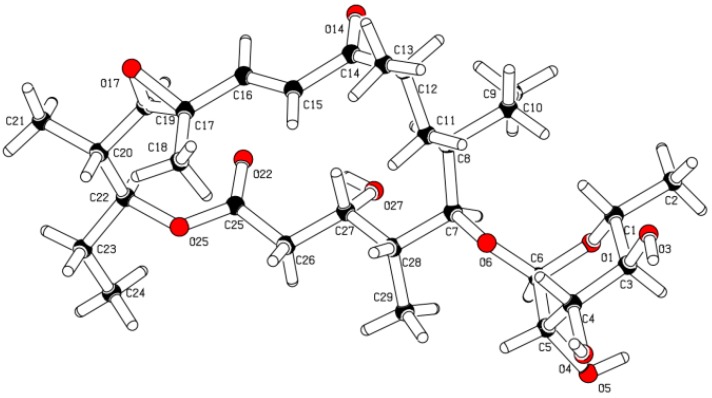
Crystal structure of **1** depicting the relative configuration.

To facilitate stereochemical determination, **1** was crystallized from methanol using a slow evaporation technique, and its relative configuration was determined by X-ray crystallographic analysis (Figure 3). We propose **1** to be the C3(*R*), C4(*S*), C5(*S*), C6(*S*), C8(*R*), C12(*S*), C13(*S*), C14(*S*), C15(*R*), C1′(*R*), C2′(*R*), C3′(*R*), C4′(*R*), C5′(*S*) enantiomer, given its stereochemical similarity to **2**. Compound **2** was isolated during the purification process of **1** and identified on the basis of HRMS, NMR, and crystallographic analysis to be 5-*O*-α-l-rhamnosyltylactone (**2**) [14] (see Supporting Information). X-ray analysis using Cu as source radiation afforded the determination of the absolute configuration of **2**. 

### 2.2. Chemopreventive Activity of **1** and **2**

Assessment of QR1 induction is used as a generic biomarker since it is known to positively correlate with the induction of other phase 2 enzymes responsible for metabolic detoxification. Phase 2 enzymes are generally regarded as being responsible for metabolic detoxification, capable of protecting an organism from oxidative damage or nucleophilic attack. Thus, induction of these enzymes can be a defensive response. Juvenimicin C (**1**) was found to induce QR1 activity with an induction ratio (IR) of 4.3 (Table 2). A more quantitative parameter, amenable for comparison of active substances, is the concentration required to double activity (CD). For compound **1**, the CD was found to be 10.1 μM. To investigate the response of compound **1** relative to compound **2**, the effect on the levels of other detoxifying enzymes and glutathione was determined. Relative to the control, **1** increased the activity of glutathione reductase (12.2 μmol NADPH oxidized/mg protein/min) and glutathione peroxidase (138 μmol NADPH oxidized/mg protein/min), and increased glutathione levels (CD = 27.7 μM). While these responses are lower than the positive control, 4′-bromoflavone [6], **1** displays activity similar to that of 2,4-dibromo-phenazine (QR1 CD = 5.9 μM, GSH CD = 20.1 μM, 12.9 μmol NADPH oxidized/mg protein/min in a glutathione reductase assay) [15], a compound which served as a scaffold for the development of inducers active in the nanomolar concentration range. Taken together, these activities demonstrate the chemopreventive potential of **1** and establish it as a target for further semi-synthetic optimization. 

**Table 2:** Chemopreventive activity of juvenimicin C (**1**) and 5-*O*-α-l-rhamnosyltylactone (**2**).

Sample	QR1 IR	QR1 CD (μM)	Glutathione CD (μM)	Glutathione reductase *	Glutathione peroxidase *
Control	1.0	na	na	9.6	63.7
1	4.3	10.1	27.7	12.2	138
2	1.4	nd	nd	nd	nd
4′-BF	8.4	0.1	5.67	18.1	154

na, not applicable; nd, not determined (**2** did not exhibit significant QR1 induction, thus further experiments were not pursued); * Reported as μmol NADPH oxidized/mg protein/min, 4′-BF (4′-bromoflavone) was used as a positive control; Control: cells treated with solvent only.

## 3. Experimental Section

### 3.1. General Experimental Procedures

Optical rotations were measured on a Perkin-Elmer 241 polarimeter. UV spectra were measured on a Shimadzu Pharma Spec UV-1700 spectrophotometer. CD spectra were acquired using a Jasco J-710 spectropolarimeter. NMR spectra were obtained on a Bruker 600 MHz DRX-600 equipped with a 1.7 mm cryoprobe and Avance III console. Chemical shifts (δ) are given in ppm and coupling constants (*J*) are reported in Hz. ^1^H and ^13^C NMR resonances of juvenimicin C (**1**) are reported in Table 1. High resolution mass spectra were obtained on an Agilent ESI-TOF spectrometer at the Scripps Center for Mass Spectrometry. Liquid chromatography mass spectrometry (LCMS) data were obtained using a Hewlett-Packard series 1100 system equipped with a reversed-phase C_18_ column (Phenomenex Luna, 100 × 4.6 mm, 5 μm) at a flow rate of 0.7 mL·min^−1^. High-performance liquid chromatography (HPLC) separations were performed using a Waters 600E system controller and pumps with a Model 480 spectrophotometer. Separation was achieved using a Phenomenex Luna semi-preparative C_18_ column (250 × 10 mm, 5 μm) with a flow rate of 2 mL·min^−1^.

### 3.2. Bacterial Isolation and Identification

Strain CNJ-878 was isolated from sediment collected off the coast of Palau using SCUBA at a depth of 25 m. Strain CNJ-878 (GenBank accession number DQ448714) shared 98.9% 16S rRNA gene sequence identity with the most closely related type strain *Micromonospora yangpuensis* (GenBank accession number GU002071) [16,17] suggesting it may represent a new species. It shared high levels of sequence identity with other marine derived strains including two *Micromonospora* spp. (CNQ-335_SD01, EU214915; CNS-633_SD06, EU214967), and 99.9% identity to three strains belonging to the proposed genus “*Solwaraspora*” (UMM543, AY552769; UMM566, AY552764; UMM483, AY552761) [18].

### 3.3. Fermentation and Extraction

Strain CNJ-878 was cultured in 39 × 1 L portions in Fernbach flasks containing high nutrient medium (filtered ocean water, 10 g starch, 4 g yeast, 2 g peptone, 1 g calcium carbonate, 100 mg potassium bromide, and 40 mg iron sulfate) for 7 day at 25 °C while shaking at 230 rpm. 

Sterilized Amberlite XAD-16 resin (20 g·L^−1^) was added to each flask to absorb the extracellular metabolites. The culture medium and resin were shaken for 6 h, filtered using cheesecloth to remove the resin, and washed with deionized water to remove salts. The resin, cell mass, and cheesecloth were extracted with acetone overnight, concentrated under vacuum, and partitioned between water and ethyl acetate. The organic layer was dried under vacuum to afford 2.5 g of extract.

### 3.4. Isolation and Characterization of Juvenimicin C (**1**) and Isolation of 5-*O*-α-l-Rhamnosyltylactone (**2**)

The crude extract was fractionated using silica gel flash column chromatography eluting with a methanol-dichloromethane (DCM) step gradient to afford seven fractions. Fraction 4 (DCM-methanol 95:5), contained the bioactive constituents, thus it was separated using C_18_ flash column chromatography eluting with 50, 80, and 100% aqueous acetonitrile. The 50% acetonitrile fraction was further separated using RP-C_18_ HPLC (2 mL·min^−1^ isocratic flow, 70% aqueous methanol) to afford 10 fractions. Fraction 2 (*t*_R_ 11.3 min, 163 mg) was separated using RP-C_18_ semi-preparative HPLC using an isocratic flow of 70% aqueous methanol to afford semi-pure **1** (*t*_R_ 11.3 min, 6.6 mg) and **2** (*t*_R_ 12.50 min, 16.0 mg). Each fraction was subsequently purified using C_18_ reversed-phase HPLC eluting with 68% aqueous methanol to yield juvenimicin C (**1**, 4.1 mg, 0.16% yield) and 5-*O*-α-l-rhamnosyltylactone (**2**, 13.4 mg, 0.54% yield).

**Juvenimicin C** **(1)**: White amorphous powder (4.1 mg). [α]^25^_D_ −16 (c 0.03, MeOH), UV (MeOH) λ_max_ (log ε) 239 (517). ^1^H NMR (600 MHz, CD_3_CN) and ^13^C NMR (150 MHz, CD_3_CN), see Table 1. HRESI-TOF MS *m*/*z* 579.3160 [M + Na]^+^ (calcd. for C_29_H_48_O_10_Na: 579.3139). Supplementary crystallographic data for **1** were deposited under accession number CCDC 915943 and can be obtained free of charge from The Cambridge Crystallographic Data Centre.

**5-*O*-α-l-Rhamnosyltylactone** **(2)**: White amorphous powder (13.4 mg). UV (MeOH) λ_max_ 240 nm. ^1^H NMR (600 MHz, CD_3_CN), see Supporting Information; HRESI-TOF MS *m*/*z* 563.3203 [M + Na]^+^ (calcd. for C_29_H_48_O_9_Na: 563.3191). 

### 3.5. Quinone Reductase 1 (QR1) Assay

This assay was modified from a previously described protocol [19]. Cultured Hepa 1c1c7 mouse hepatoma cells were plated at a density of 2 × 10^4^ cells·mL^−1^ in 96-well plates and incubated for 24 h. The medium was then changed, and test compounds, dissolved in 10% dimethyl sulfoxide (DMSO), were introduced and serially diluted to a concentration range of 0.15–20 μg·mL^−1^. The cells were incubated for an additional 48 h. Quinone reductase activity was measured by the NADPH-dependent menadiol-mediated reduction of 3-(4,5-dimethylthiazo-2-yl)-2,5-diphenyltetrazolium bromide (MTT) to a blue formazan.

Normalization to protein levels was accomplished using crystal violet staining of duplicate plates, and subsequent measurement at 595 nm [20]. Enzyme activity was expressed as a CD value, the concentration of test material needed to double the specific activity of quinone reductase (reported in micromolar). The known QR1 inducer 4′-bromoflavone (CD = 0.1 μM) was used as a positive control [6].

### 3.6. Determination of GSH Levels in Cell Culture

Glutathione was measured via oxidation of 5,5-dithiobis-(2-nitrobenzoic acid) (Ellman reagent) and reduction by NADPH in the presence of glutathione reductase, as previously described [21]. Briefly, Hepa 1c1c7 cells were seeded in 96-well plates (200 μL/well) at a concentration of 2 × 10^4^ cells·mL^−1^. Following a 24 h incubation, five serial dilutions of test compounds in 0.5% DMSO (final concentration) and fresh medium were added in duplicate. Plates were further incubated for 48 h, washed three times with PBS (pH 7.4), and frozen at −80 °C. Cells were lysed by three consecutive freeze-thaw cycles, followed by the addition of 40 μL of 125 μM sodium phosphate buffer (pH 7.5) containing 6.3 mM EDTA (solution A). A reaction mixture was prepared consisting of 20 μL of 6 mM 5,5-dithiobis-(2-nitobenzoic acid) in solution A, 10 μL of glutathione reductase solution (50 units in 10 mL solution A), and 140 μL of NADPH-generating system (solution B). Solution B contained 2.5 mL of 0.5 M Tris-HCl (pH 7.4), 330 μL of 150 mM glucose 6-phosphate, 100 units of glucose 6-phosphate dehydrogenase, and 30 μL of 50 mM NADP^+^ in a total volume of 50 mL distilled water. Freshly prepared reaction mixture (170 μL) was added to each well and plates were shaken at room temperature for five minutes. After five minutes of further incubation, the formation of 2-nitro-5-thiobenzoic acid was measured at 405 nm. Protein content was measured using a bicinchoninic acid protein assay kit with BSA as a standard.

### 3.7. Determination of Glutathione Reductase Activity

To measure the activity of glutathione reductase, cell lysates were added to a mixture containing 1.78 mM EDTA in 178 mM potassium phosphate buffer, 1 mM glutathione disulfide (GSSG), and 0.1 mM NADPH in 10 mM Tris-HCl in a final volume of 1 mL. The linear decrease in absorbance of NADPH was measured at 340 nm for 2 min. Activity was normalized per mg protein [22].

### 3.8. Determination of Glutathione Peroxidase Activity

Glutathione peroxidase activity was measured by combining tissue supernatant and 31.5 mM sodium phosphate buffer, 1 mM GSH, 0.2 mM β-NADPH, 11.25 mM sodium azide, and 10 units glutathione reductase in a final volume of 1.0 mL. After allowing the incubation mixture to equilibrate for a few minutes, 0.238 mM hydrogen peroxide was added. The linear decrease in absorbance of NADPH was measured at 340 nm for 1 min following a lag time of 30 s. Activity was normalized per mg protein [22].

## 4. Conclusions

We identified a new macrolide glycoside, juvenimicin C (**1**), from a marine-derived *Micromonospora* sp. Compound **1** enhanced QR1 enzyme activity and glutathione levels by two-fold with CD values of 10.1 and 27.7 μM, respectively. In addition, glutathione reductase and glutathione peroxidase activities were elevated. Additionally γ,δ-unsaturated analog (**2**) was isolated yet not active. This is the first reported member of the macrolide class of antibiotics found to mediate these responses. 

## Supplementary Files

Supplementary File 1 Supporting Information (PDF, 773 KB)
