# Supplementary material for: Potential Chemopreventive Activity of a New Macrolide Antibiotic from a Marine-Derived Micromonospora sp
Source: Mar Drugs. 2013 Apr 3;11(4):1152–61. doi: 10.3390/md11041152 (PMC3705395; doi:10.3390/md11041152)

Supporting Information

Table of Contents

Figure S1. ^{1}H NMR spectrum (500 MHz) of juvenimicin C (**1**) in acetonitrile-d_3.
Figure S2. COSY spectrum (500 MHz) of juvenimicin C (**1**) in acetonitrile-d_3.
Figure S3. HMBC spectrum (500 MHz) of juvenimicin C (**1**) in acetonitrile-d_3.
Figure S4. NOESY spectrum (500 MHz) of juvenimicin C (**1**) in acetonitrile-d_3.
Figure S5. Selective 1D-TOCSY spectrum of H2 (500 MHz) of juvenimicin C (**1**) in acetonitrile-d_3.
Figure S6. Selective 1D-TOCSY spectrum of H3 (500 MHz) of juvenimicin C (**1**) in acetonitrile-d_3.
Figure S7. Selective 1D-TOCSY spectrum of H8 (500 MHz) of juvenimicin C (**1**) in acetonitrile-d_3.
Figure S8. Selective 1D-TOCSY spectrum of H15 (500 MHz) of juvenimicin C (**1**) in acetonitrile-d_3.
Figure S9. Selective 1D-TOCSY spectrum of H18 (500 MHz) of juvenimicin C (**1**) in acetonitrile-d_3.
Figure S10. Selective 1D-TOCSY spectrum of H1′ (500 MHz) of juvenimicin C (**1**) in acetonitrile-d_3.
Figure S11. Selective 1D-TOCSY spectrum of H5′ (500 MHz) of juvenimicin C (**1**) in acetonitrile-d_3.
Figure S12. TOCSY spectrum (500 MHz) of juvenimicin C (**1**) in acetonitrile-d_3.
Figure S13. Expanded HR-ESI-FT-MS of juvenimicin C (**1**).
Figure S14. CD spectrum of juvenimicin C (**1**) in methanol.
Figure S15. UV spectrum of juvenimicin C (**1**) in methanol.
Figure S16. ^{1}H-NMR spectrum of 5-*O*-α-L-rhamnosyltylactone (**2**).
Figure S17. HR-ESI-FT-MS of 5-*O*-α-L-rhamnosyltylactone (**2**).
Figure S18. Crystal structure of 5-*O*-α-L-rhamnosyltylactone (**2**).

Figure S1. ^{1}H NMR spectrum (500 MHz) of juvenimicin C (**1**) in acetonitrile-d_3.

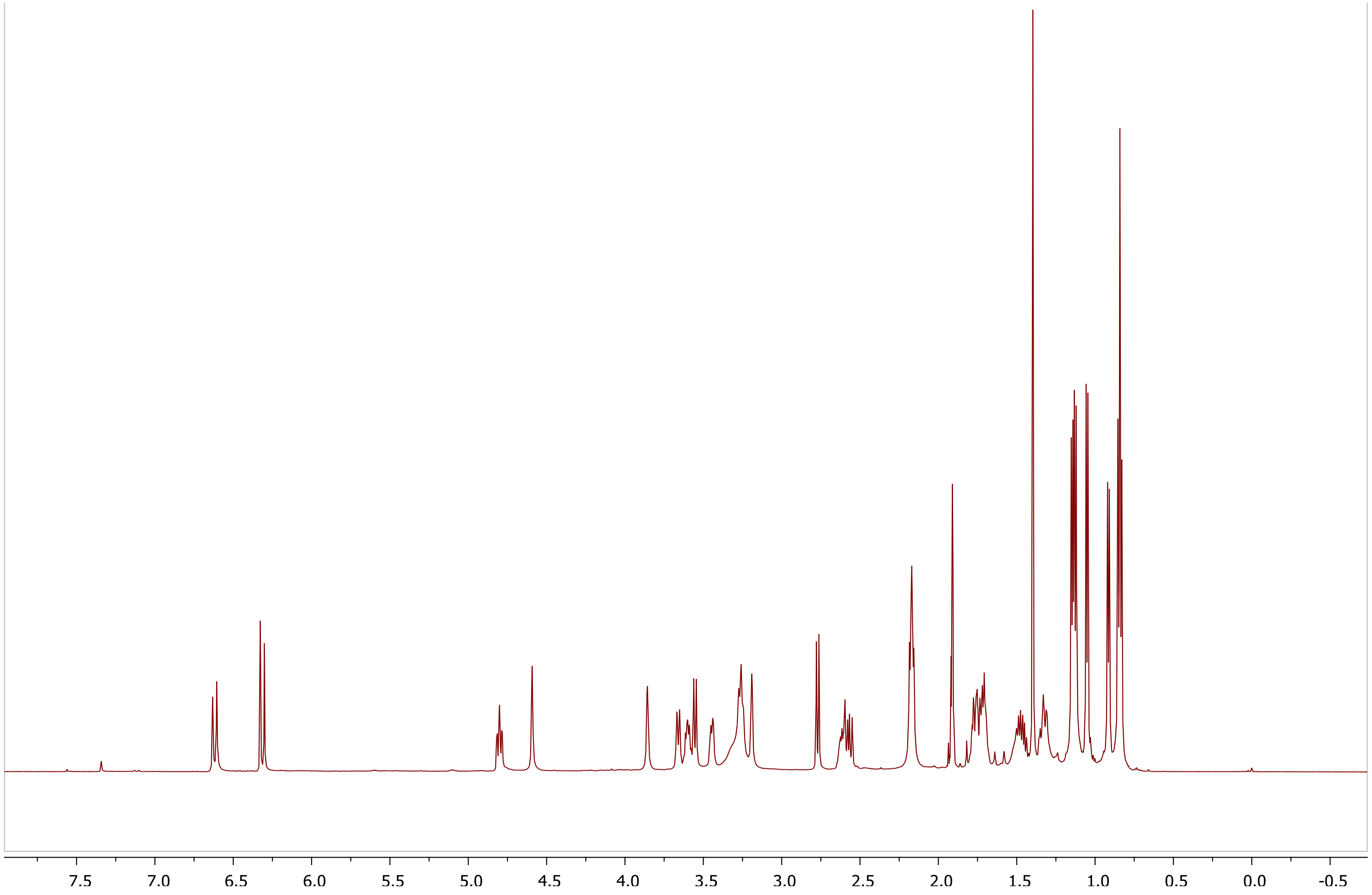

Figure S2. COSY spectrum (500 MHz) of juvenimicin C (**1**) in acetonitrile-d_3.

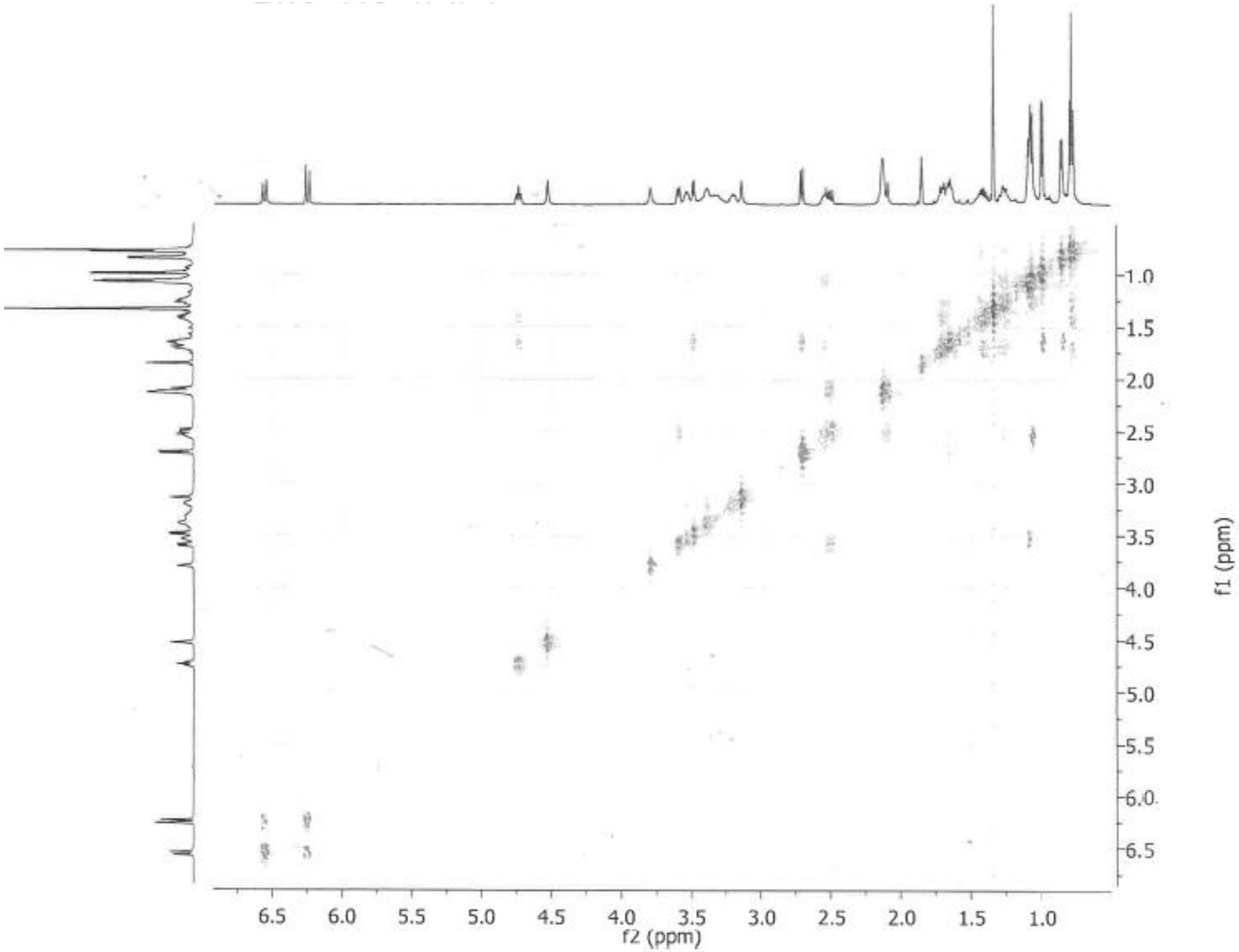

Figure S3. HMBC spectrum (500 MHz) of juvenimicin C (**1**) in acetonitrile-d_3.

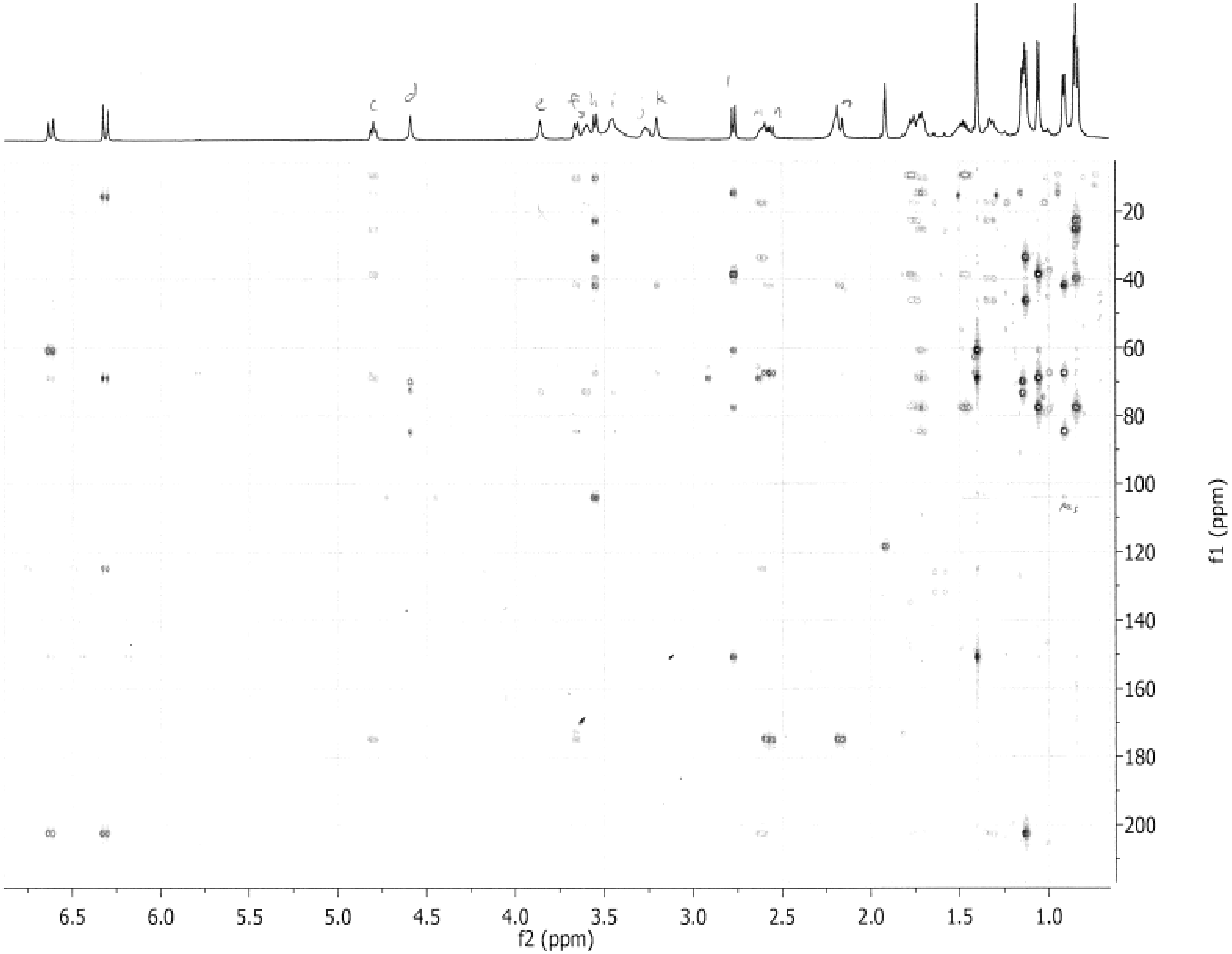

Figure S4. NOESY spectrum (500 MHz) of juvenimicin C (**1**) in acetonitrile-d_3.

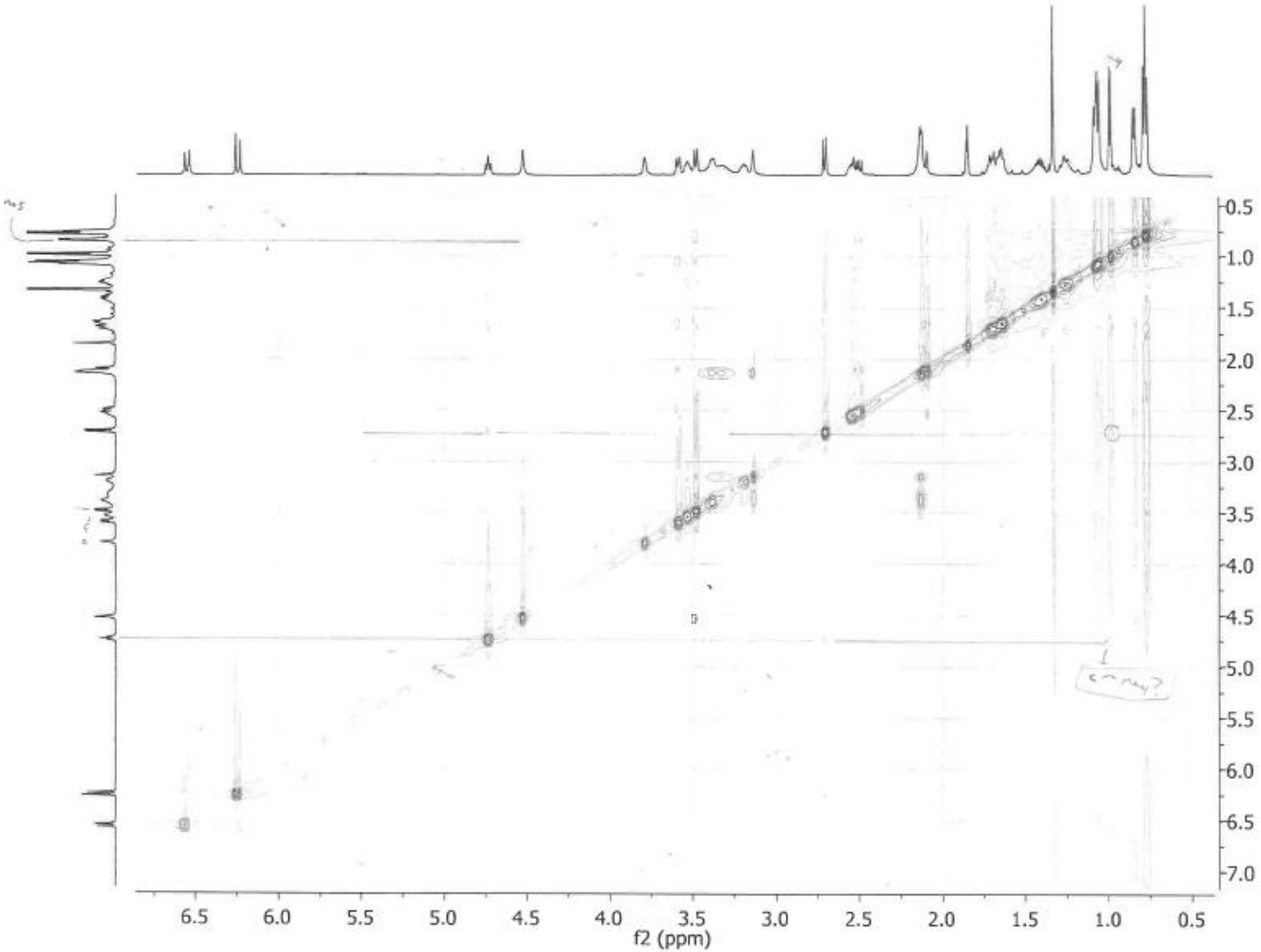

Figure S5. Selective 1D-TOCSY spectrum of H2 (500 MHz) of juvenimicin C (**1**) in acetonitrile-d_3.

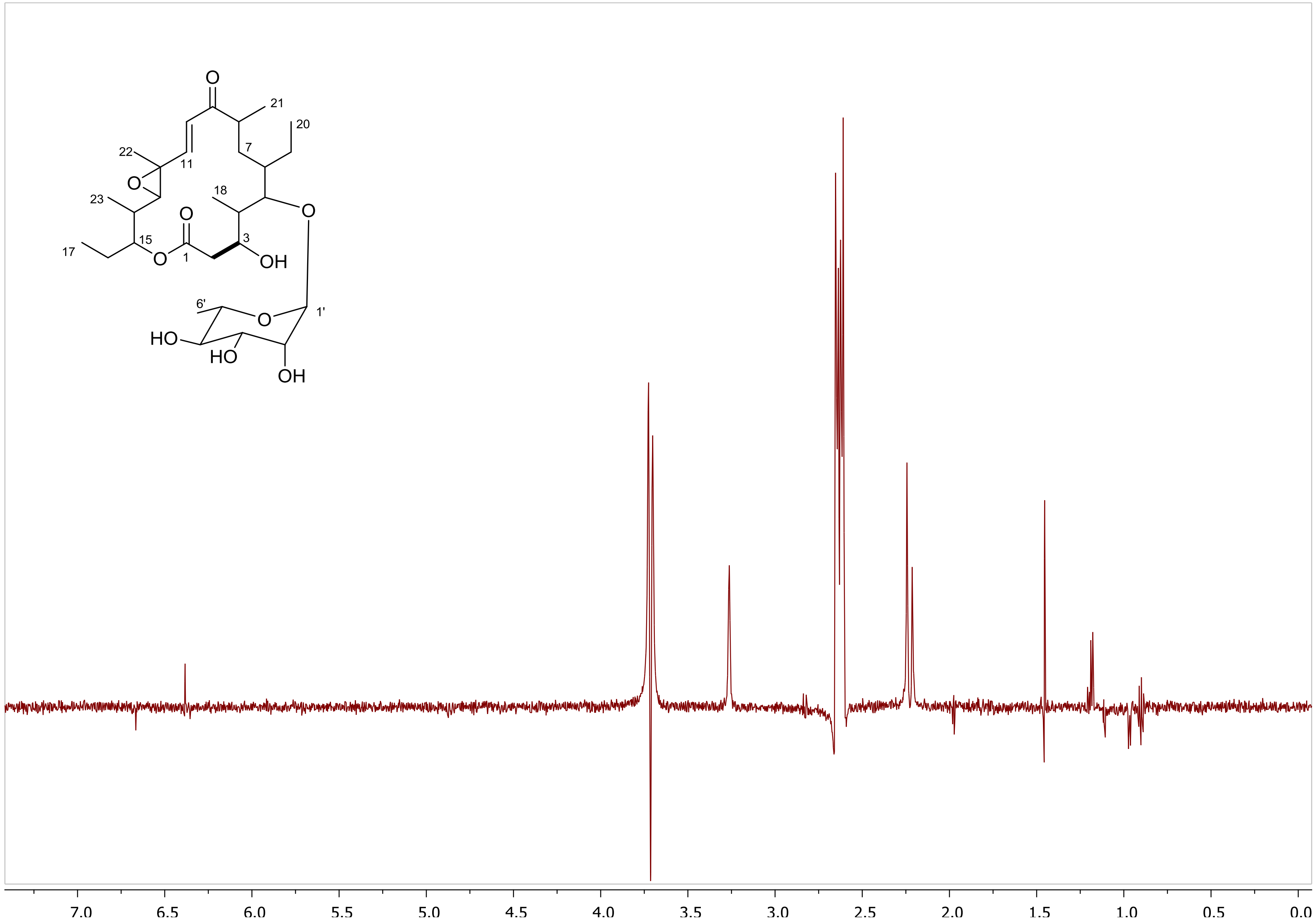

Figure S6. Selective 1D-TOCSY spectrum of H3 (500 MHz) of juvenimicin C (**1**) in acetonitrile-d_3.

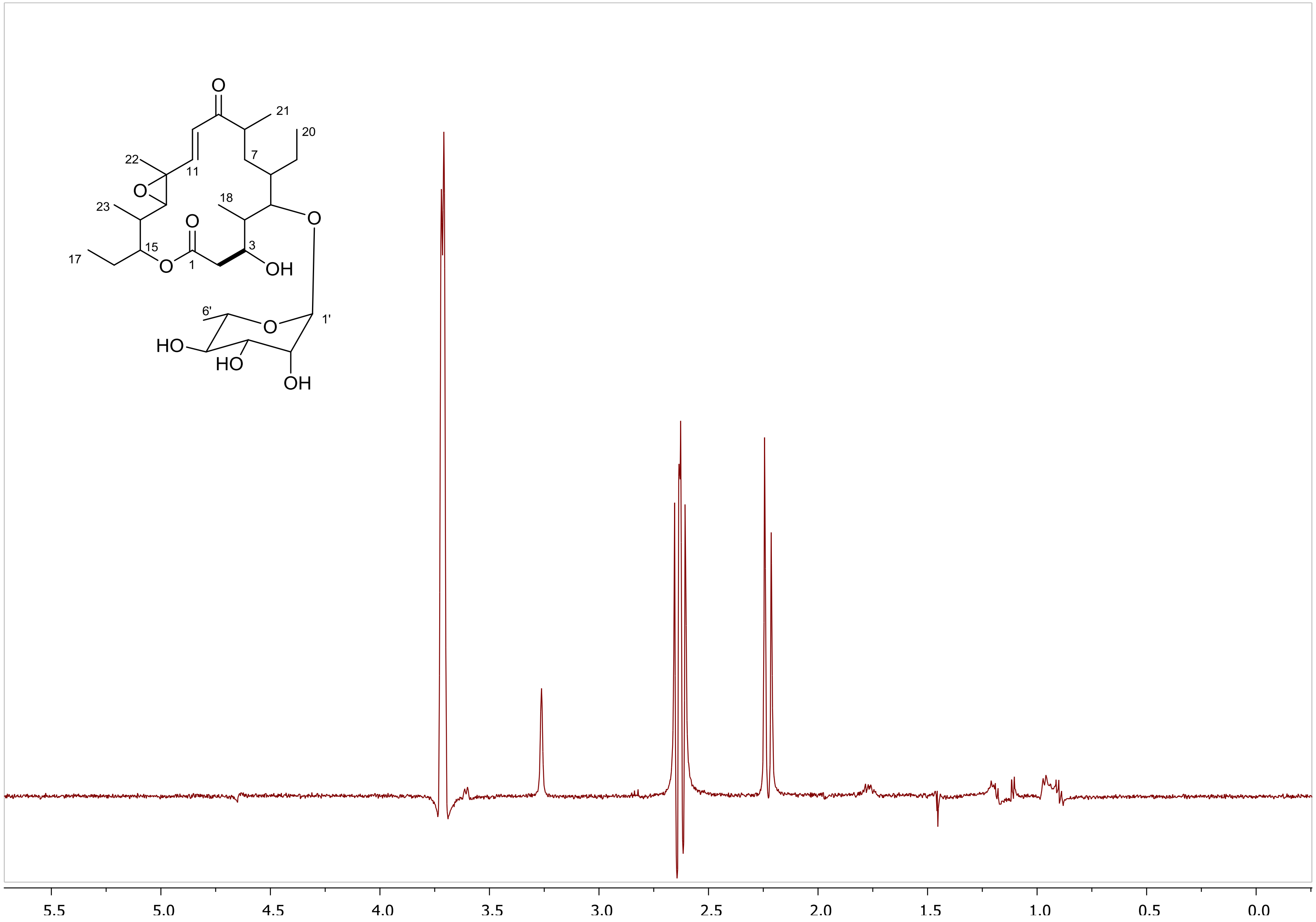

Figure S7. Selective 1D-TOCSY spectrum of H8 (500 MHz) of juvenimicin C (**1**) in acetonitrile-d_3.

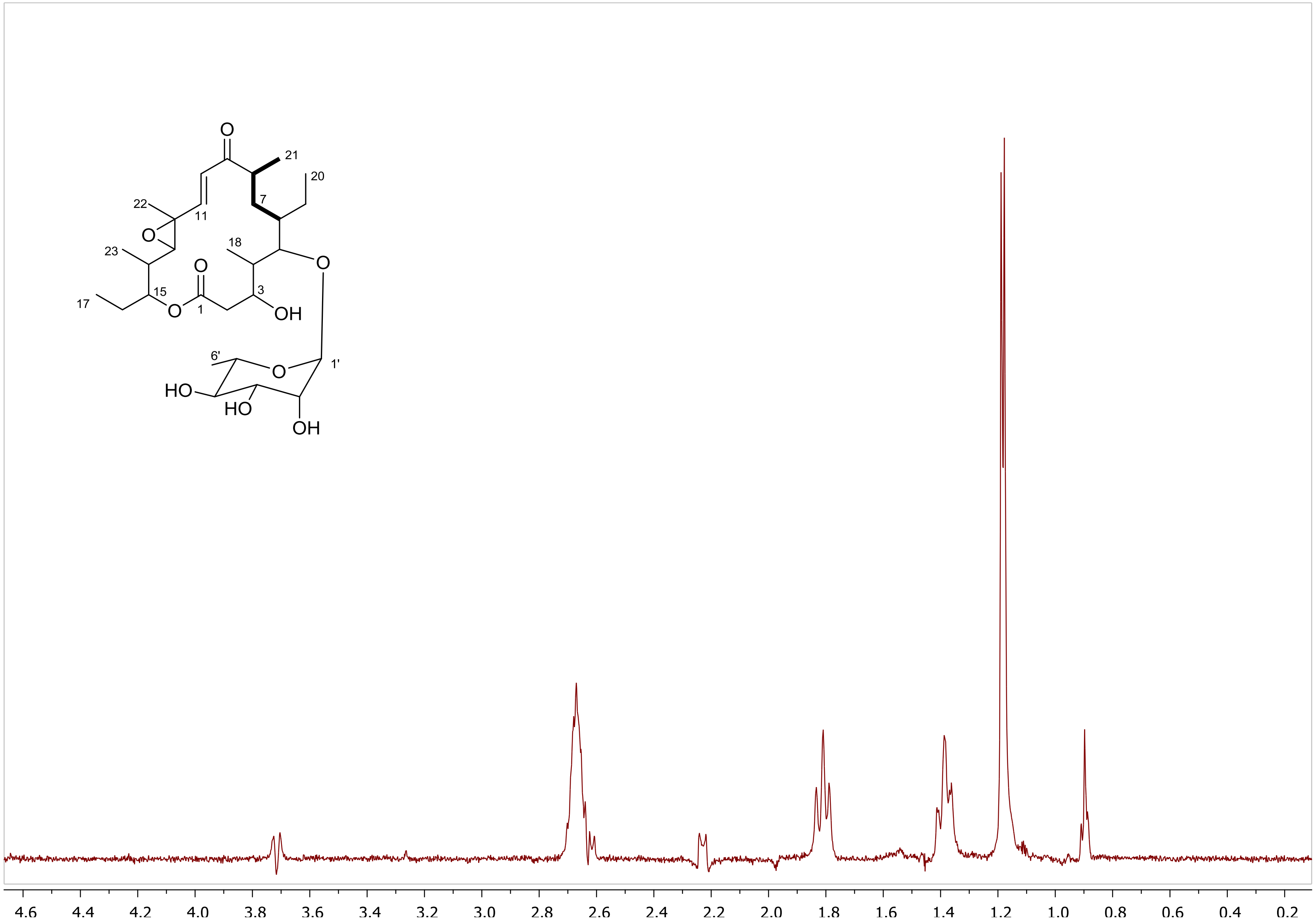

Figure S8. Selective 1D-TOCSY spectrum of H15 (500 MHz) of juvenimicin C (**1**) in acetonitrile-d_3.

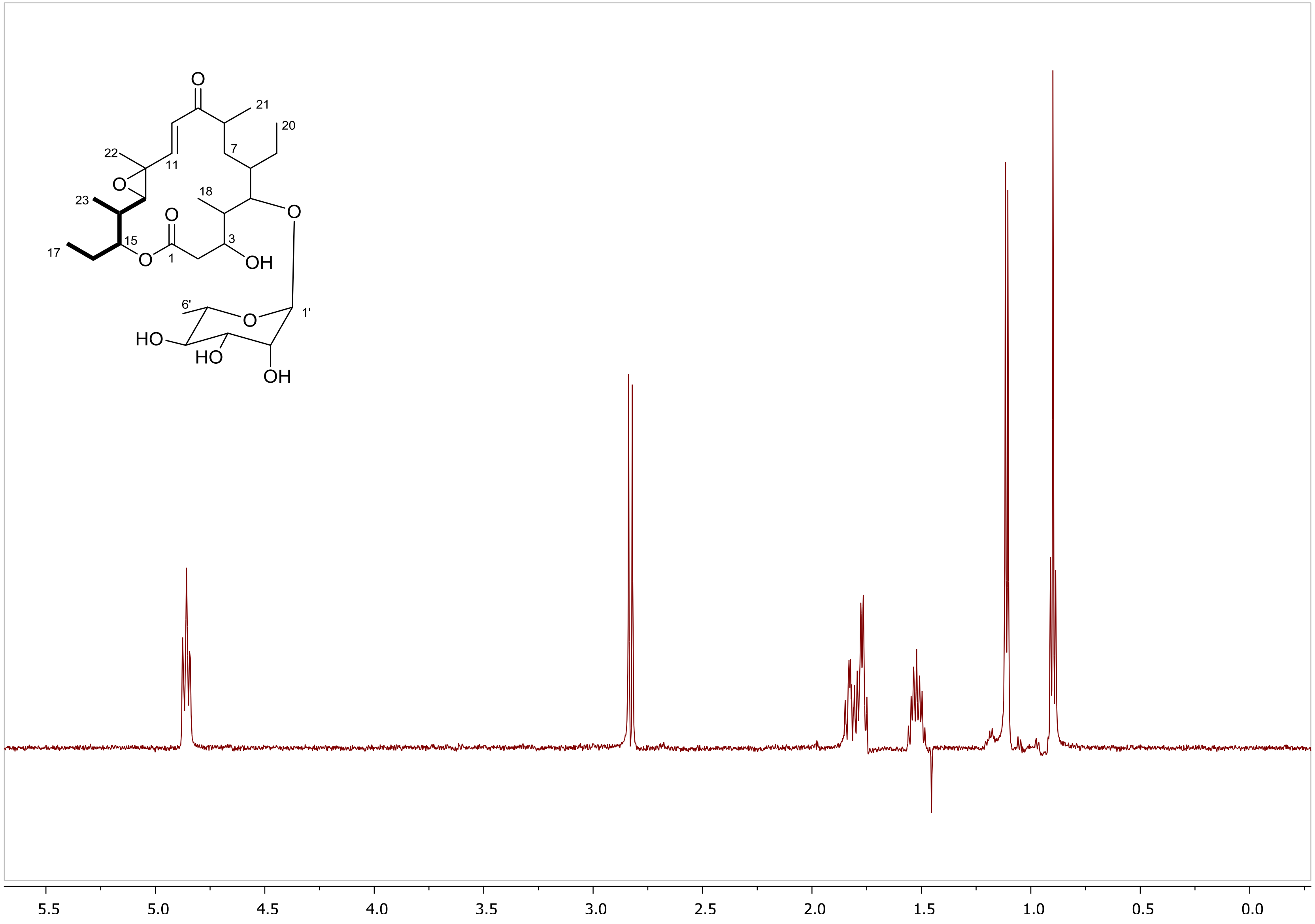

Figure S9. Selective 1D-TOCSY spectrum of H18 (500 MHz) of juvenimicin C (**1**) in acetonitrile-d_3.

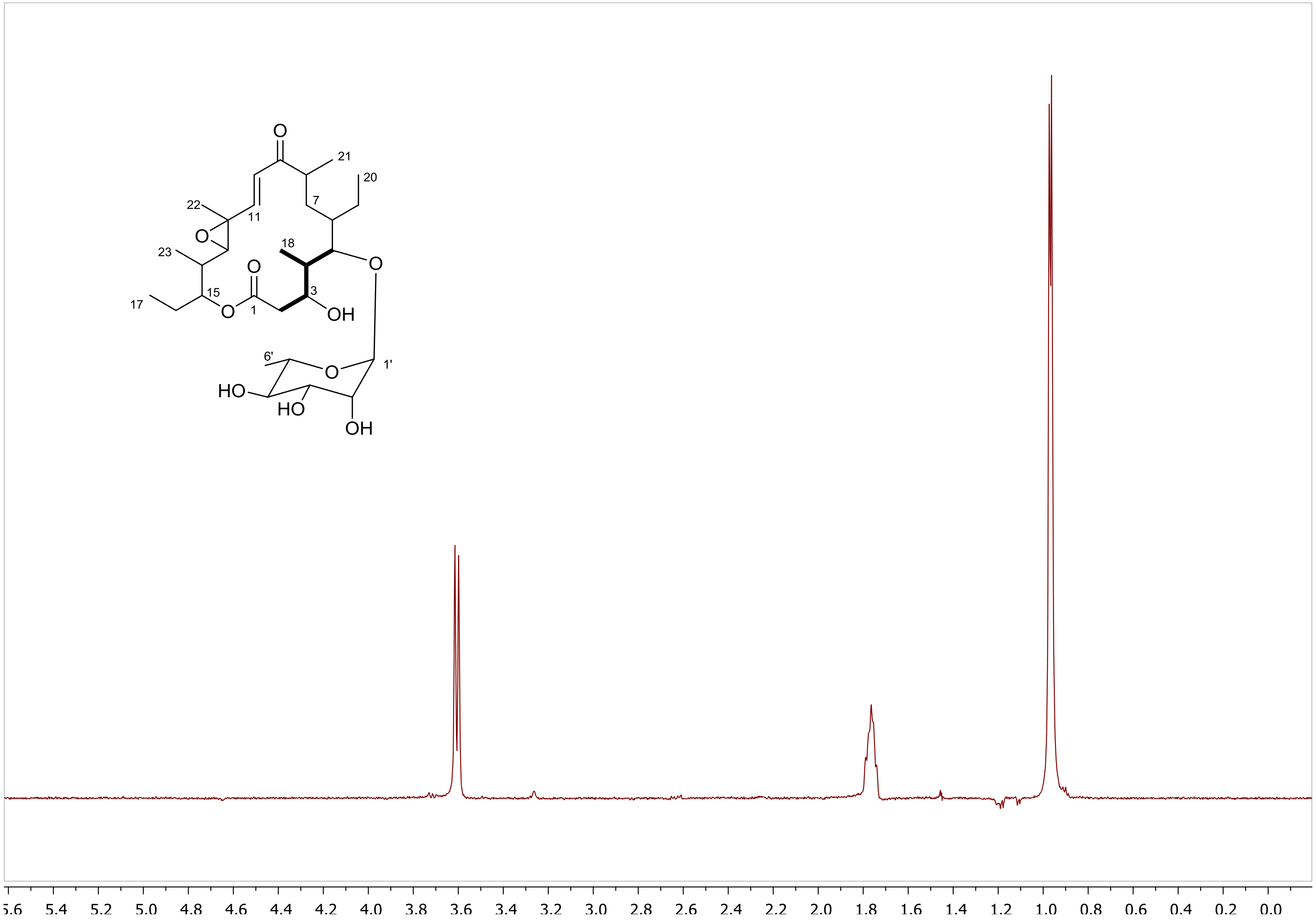

Figure S10. Selective 1D-TOCSY spectrum of H1′ (500 MHz) of juvenimicin C (**1**) in acetonitrile-d_3.

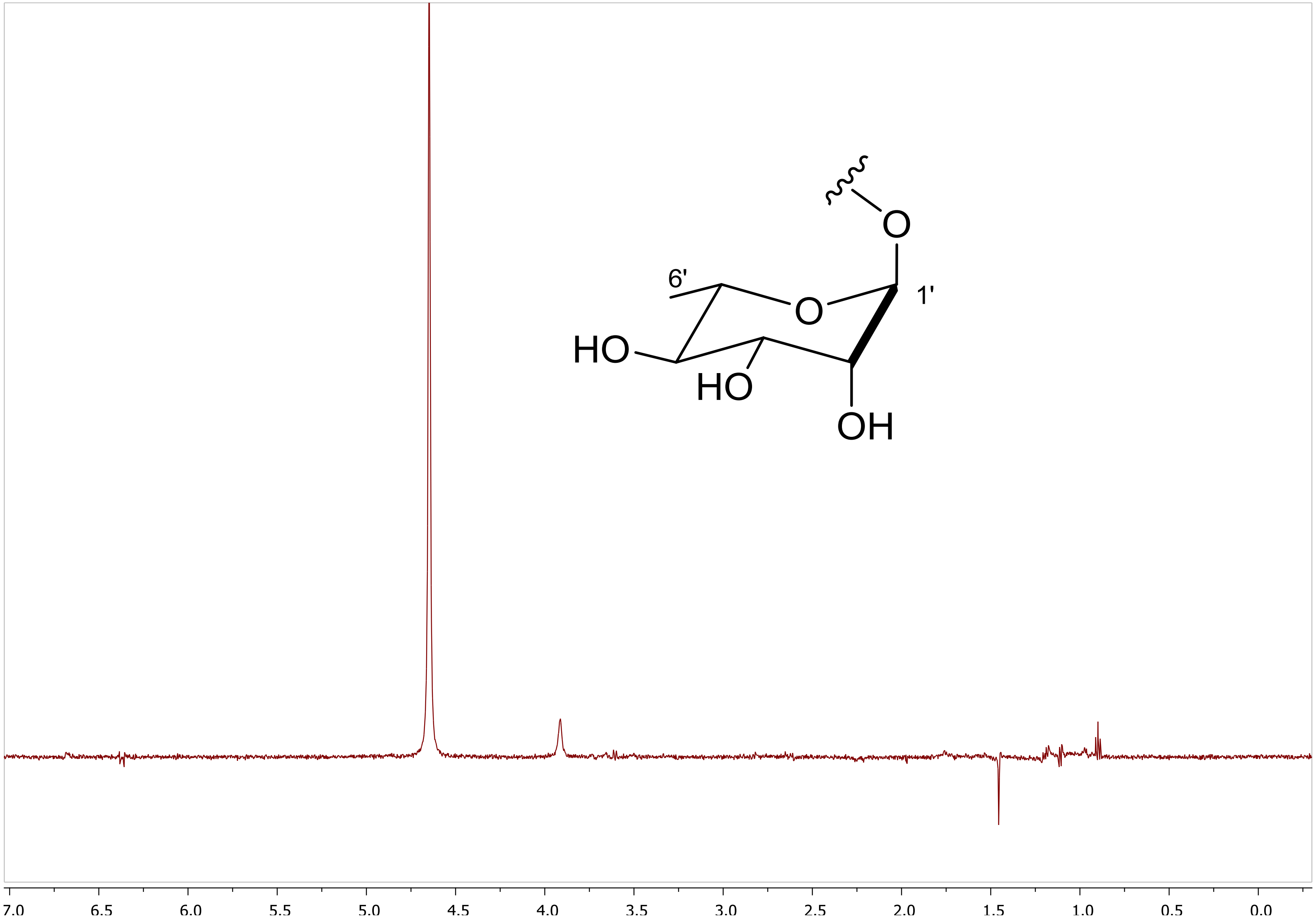

Figure S11. Selective 1D-TOCSY spectrum of H5′ (500 MHz) of juvenimicin C (**1**) in acetonitrile-d_3.

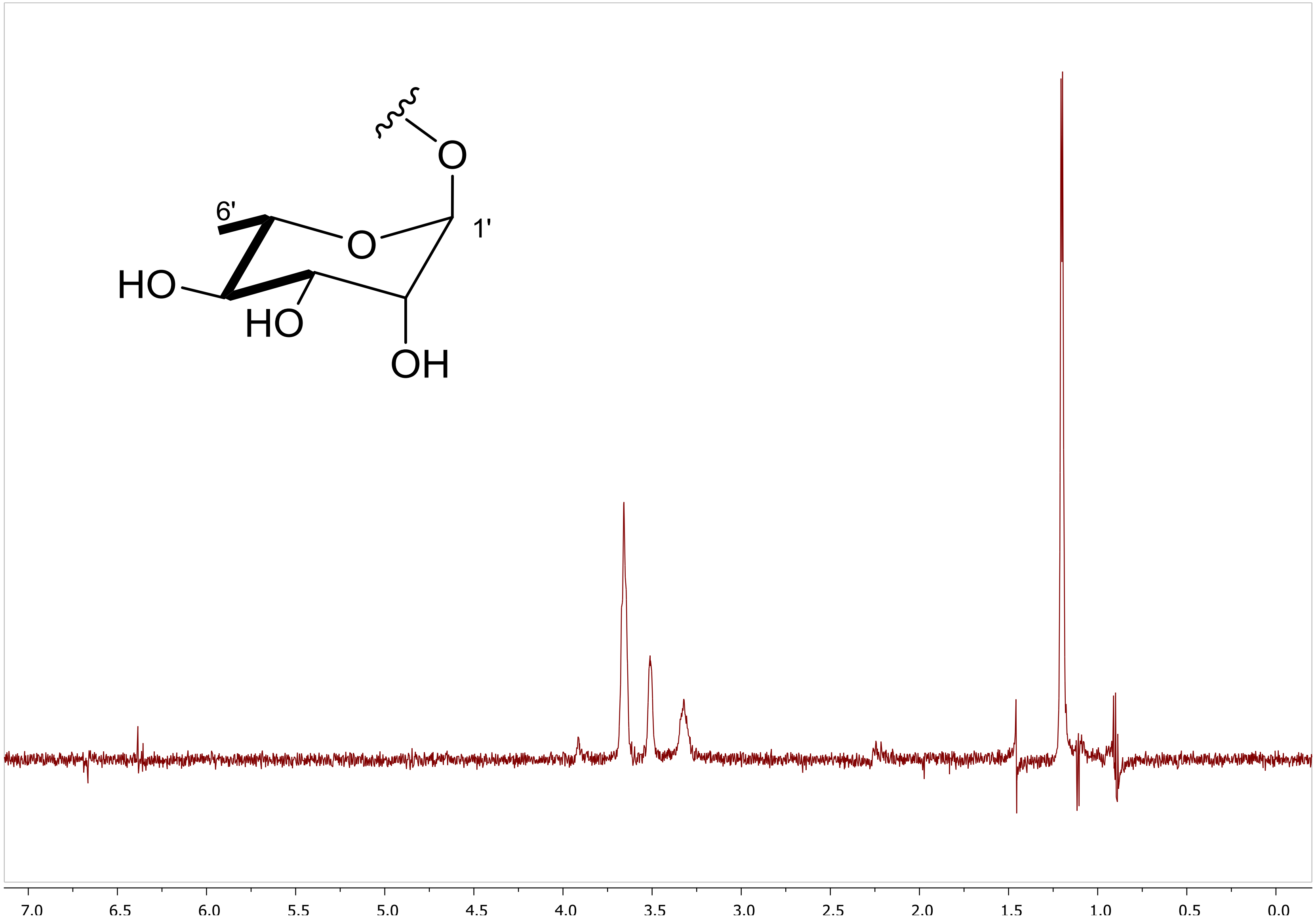

Figure S12. TOCSY spectrum (500 MHz) of juvenimicin C (**1**) in acetonitrile-d_3.

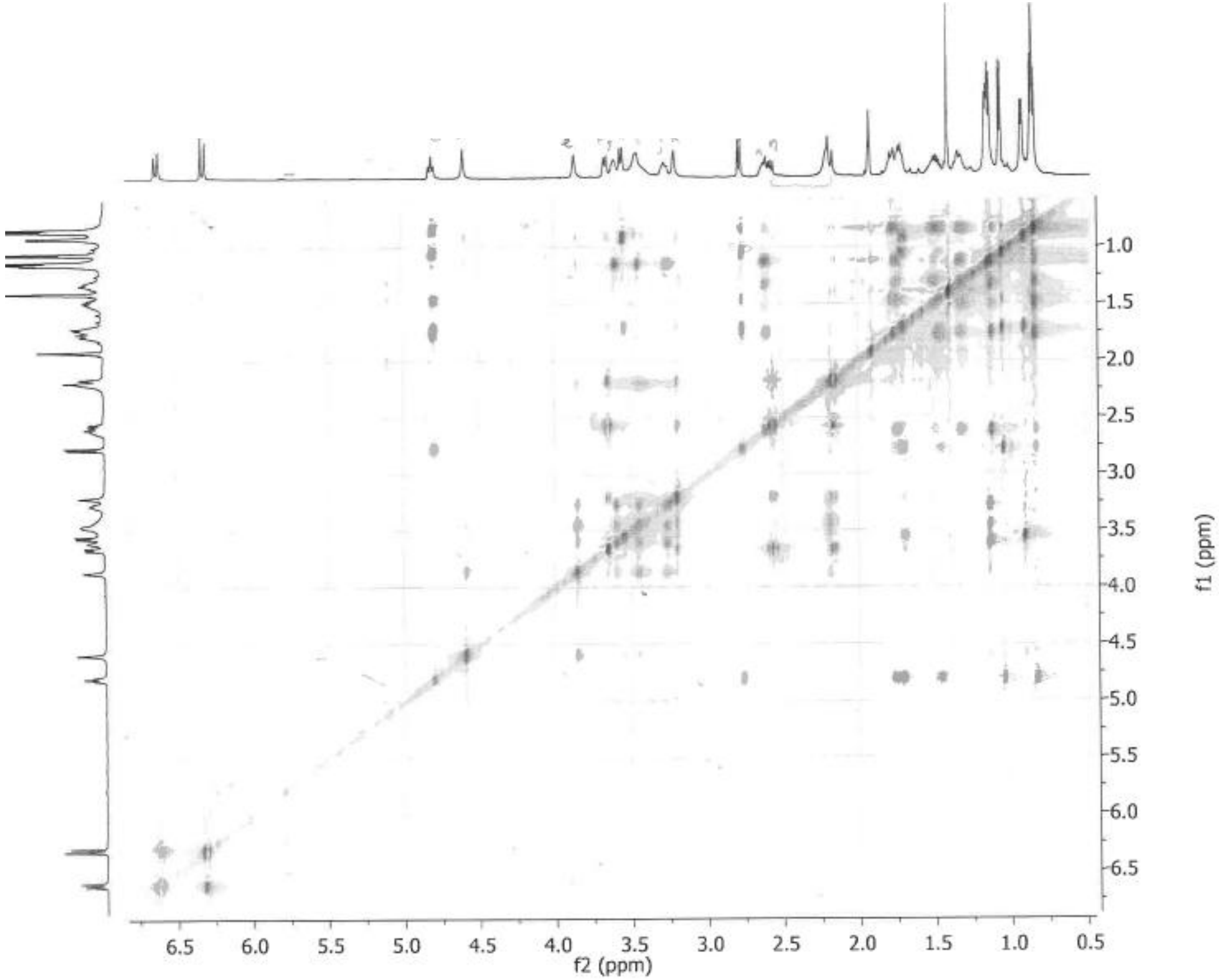

Figure S13. Expanded HR-ESI-FT-MS of juvenimicin C (**1**).

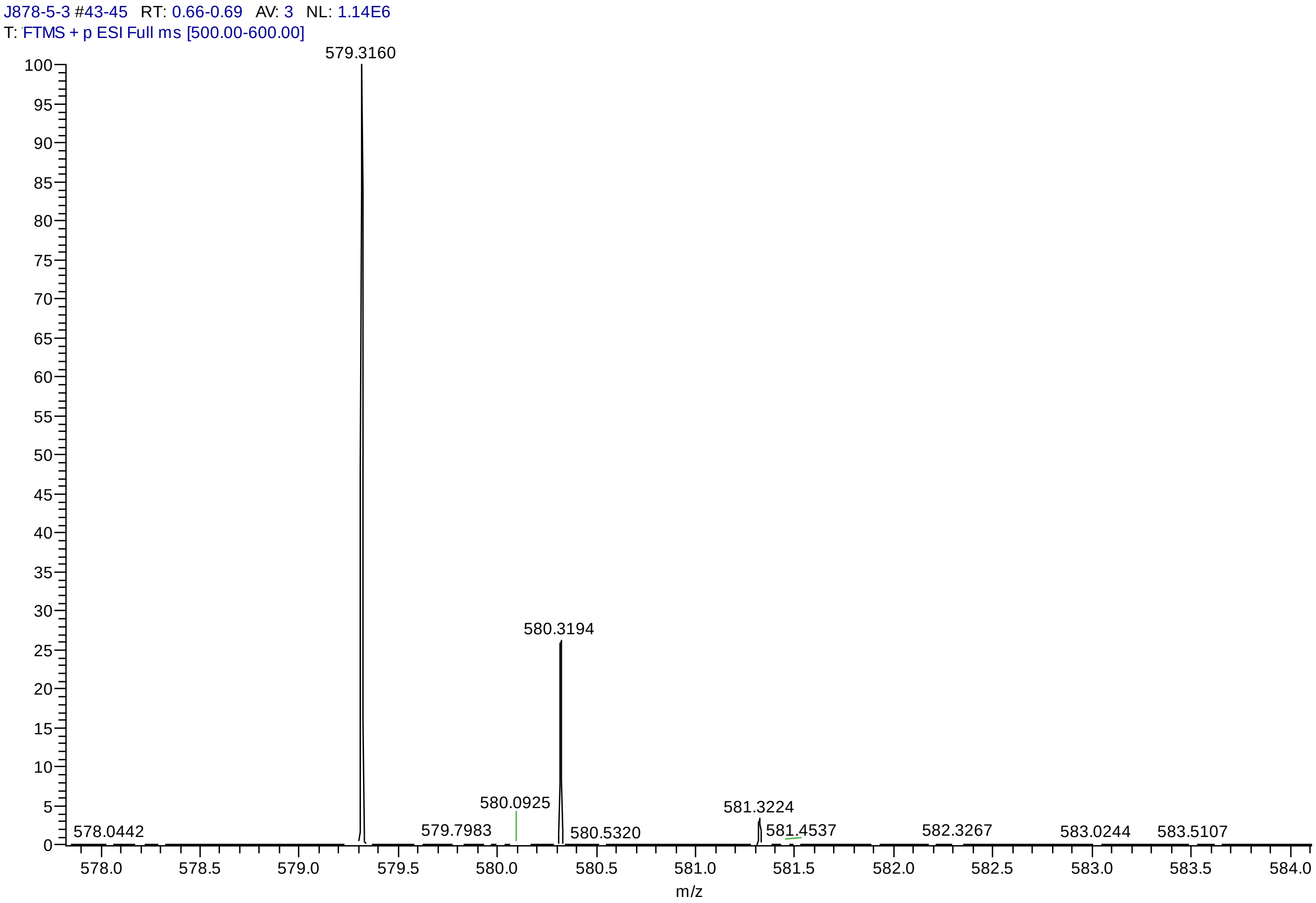

Figure S14. CD spectrum of juvenimicin C (**1**) in methanol.

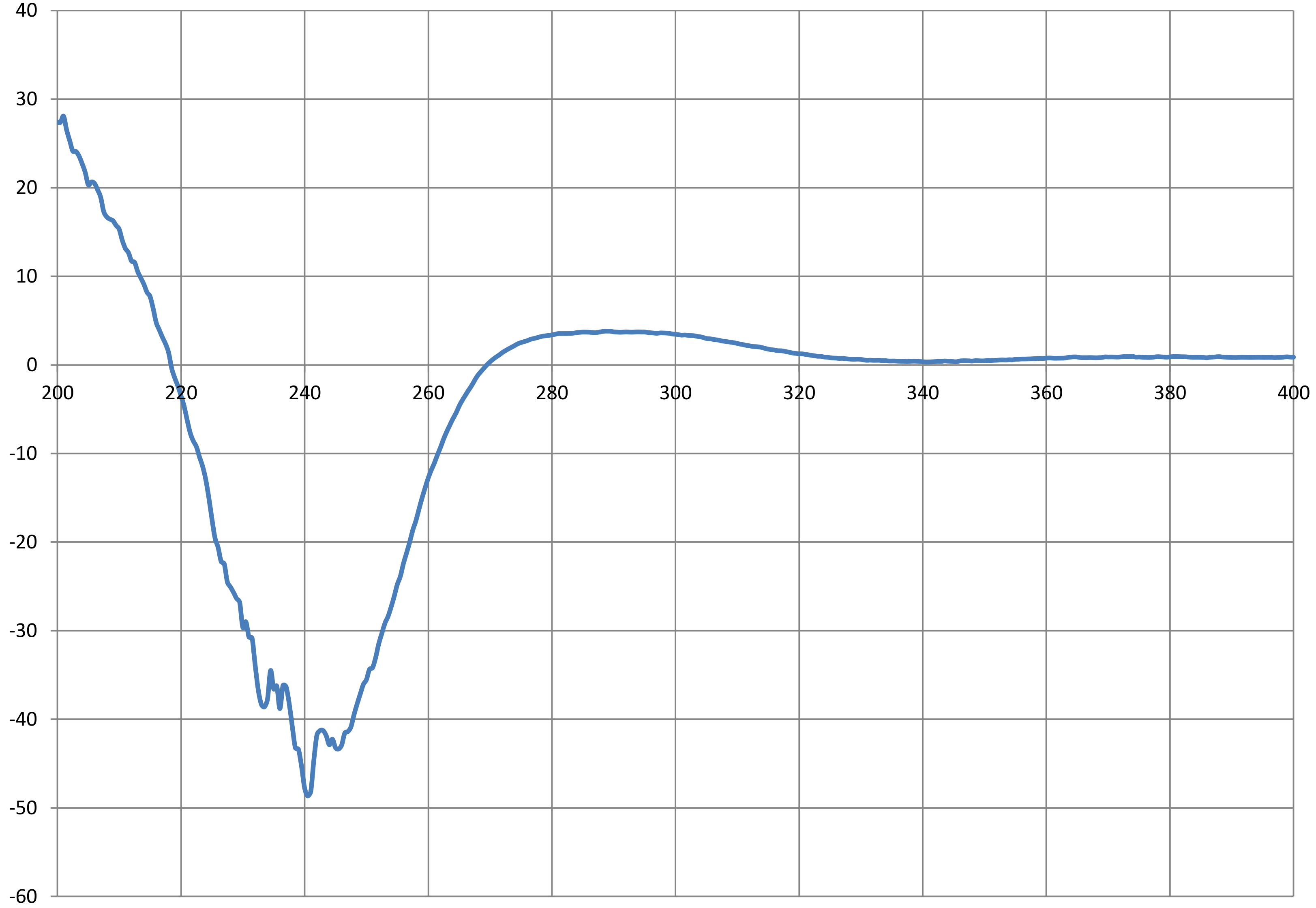

Figure S15. UV spectrum of juvenimicin C (**1**) in methanol.

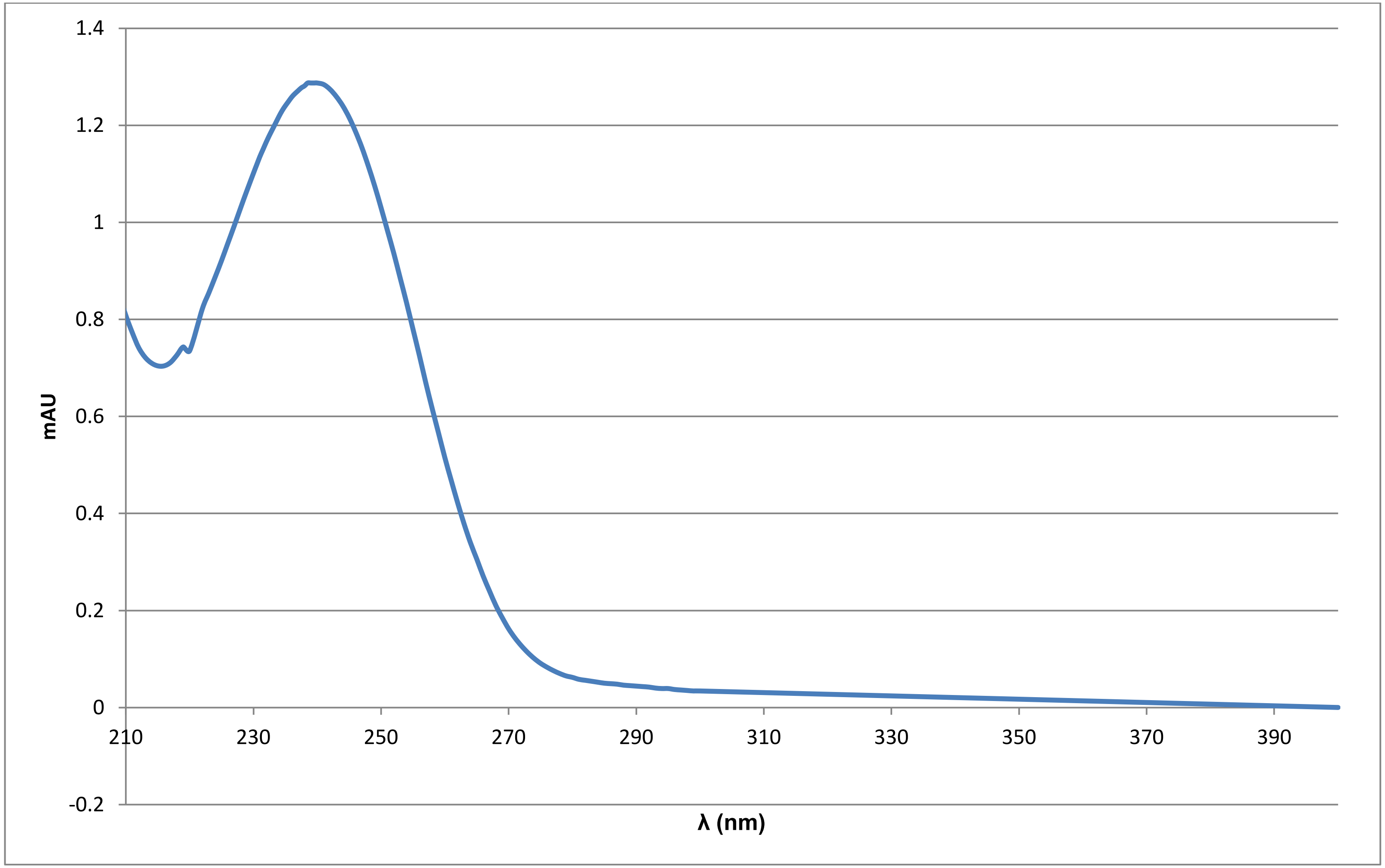

Figure S16. ^{1}H-NMR of 5-*O*-α-L-rhamnosyltylactone (**2**) in acetonitrile-d_3.

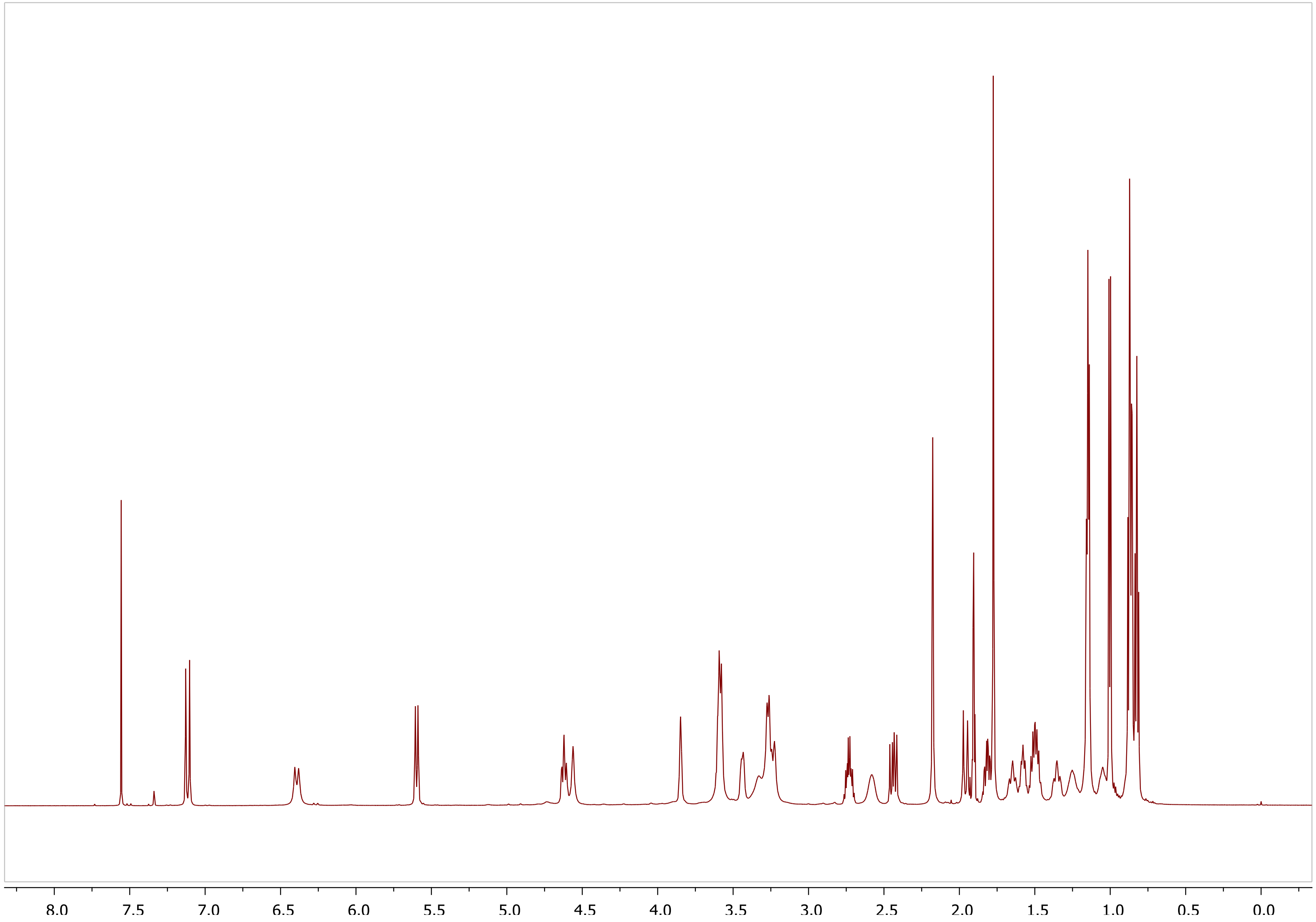

Figure S17. HR-ESI-FT-MS of 5-*O*-α-L-rhamnosyltylactone (**2**).

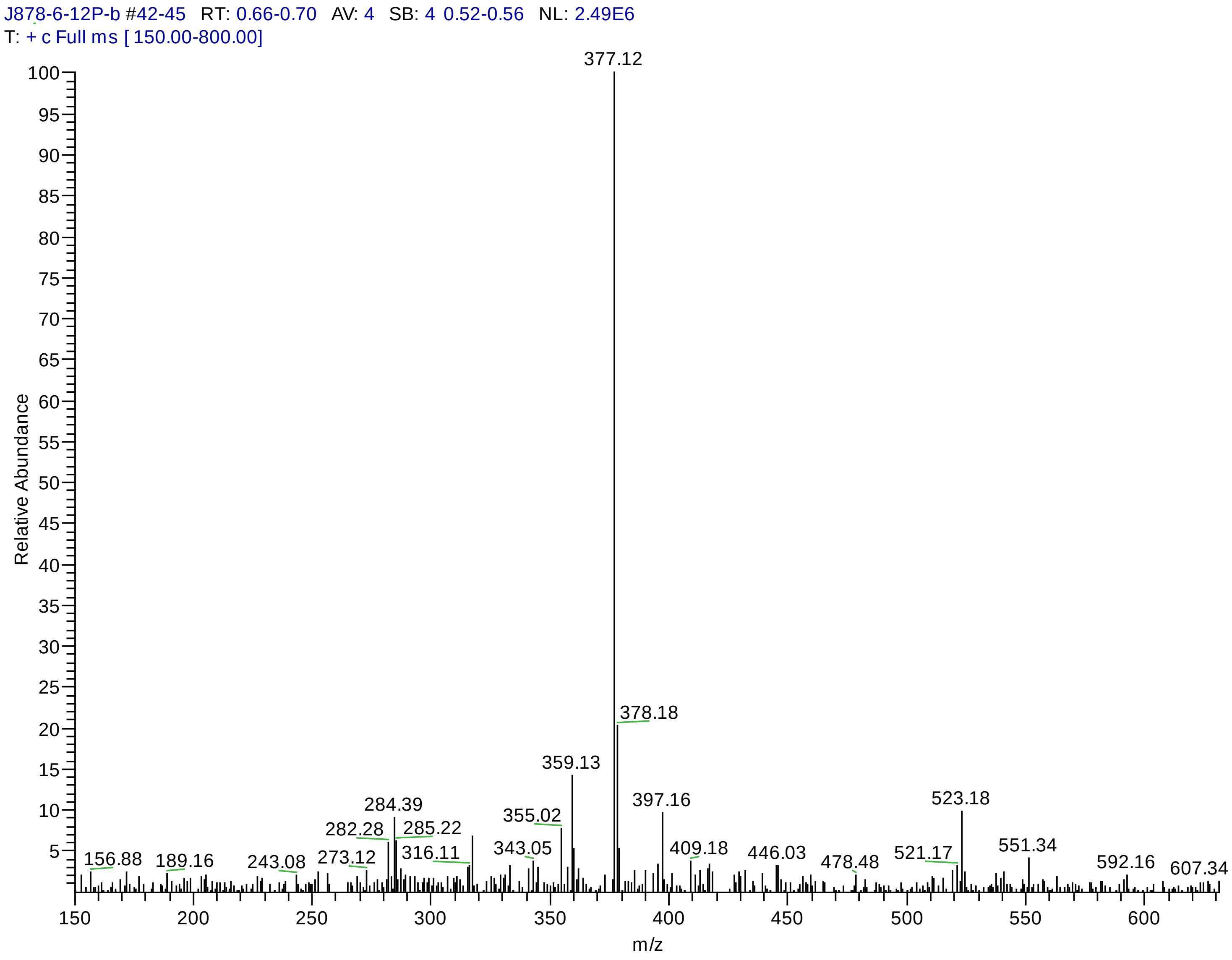

Figure S18. Crystal structure of 5-*O*-α-L-rhamnosyltylactone (**2**) depicting absolute stereochemistry.

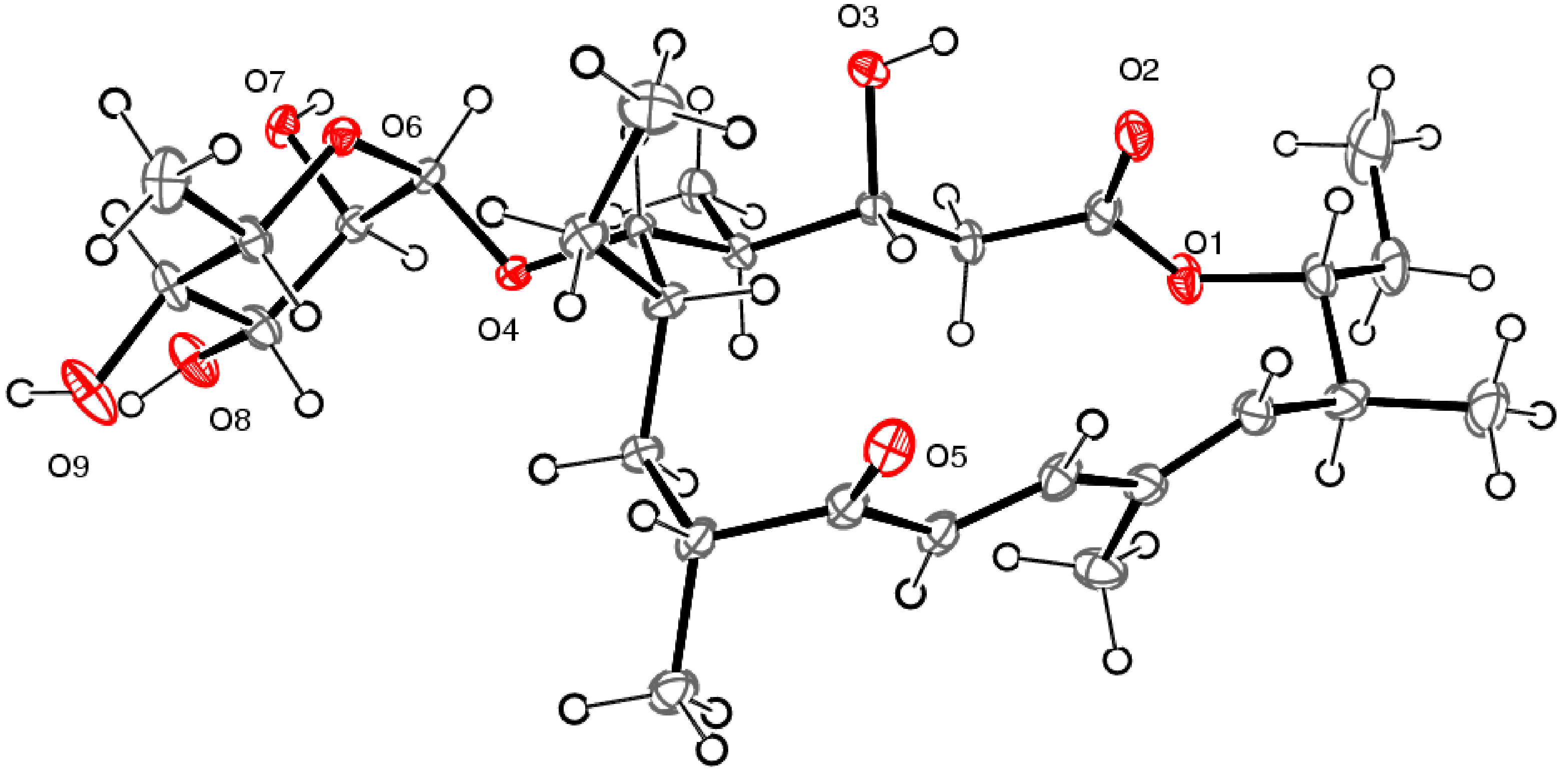

Supplement: Supplementary File 1 — Supporting Information (PDF, 773 KB) [file marinedrugs-11-01152-s001.pdf]
